# Laparoscopic Ureterocalicostomy Technique

**DOI:** 10.1590/S1677-5538.IBJU.2022.0521

**Published:** 2023-03-31

**Authors:** Romulo S. S. Nunes, Caio V. Suartz, Hiury S. Andrade, Ricardo D. Jordão, Victor Srougi, Anuar I. Mitre, William C. Nahas, Marco A. Arap

**Affiliations:** 1 Universidade de São Paulo Departamento de Videolaparoscopia em Urologia São Paulo SP Brasil Departamento de Videolaparoscopia em Urologia, Universidade de São Paulo - USP, São Paulo, SP, Brasil

## Abstract

**Purpose:**

Ureterocalicostomy is a technique that was first described by Neuwirt in 1948 (1) The laparoscopic access was initiated in 2003 by Cherullo et al. (2), following the established principles of open surgery. In 2004, Gill et al. had two patients with UPJO treated with laparoscopic ureterocalicostomy, with success (3). In 2014, Arap et. al. presented a case series with good results in adults and children in our service (4). There are factors that prepare the surgeon for an ureterocalicostomy, such as the renal cortex thickness, although the decision is mainly taken during the procedure (5).

**Material and Methods:**

A 24 years-old female patient with right lumbar pain was referred to our institution. She already had a right open pyeloplasty two years ago. The CT scan presented a right hydronephrotic kidney, DMSA scan with 30% of relative function and a DTPA scan with an obstructive pattern.

**Results:**

A laparoscopic ureterocalicostomy was performed due to the intra-operative findings (inferior kidney pole thickness and challenging access to the uretero-pelvic junction). The overall time was 130 minutes with no complications. The patient was discharged in two days and the double J was withdrawn in four weeks. The CT scan within one year demonstrates a reduction of the hydronephrosis. She had no more lumbar pain.

**Conclusion:**

In complex cases, the laparoscopic ureterocalicostomy proves to be a safe and efficient procedure, with a free tension-free anastomosis and the advantages of the laparoscopic access.
